# TLR2 Mediates *Helicobacter pylori*–Induced Tolerogenic Immune Response in Mice

**DOI:** 10.1371/journal.pone.0074595

**Published:** 2013-09-13

**Authors:** Xia Sun, Min Zhang, Mohamad El-Zataari, Stephanie Y. Owyang, Kathryn A. Eaton, Maochang Liu, Yu-Ming Chang, Weiping Zou, John Y. Kao

**Affiliations:** 1 Department of Pharmacology, School of Medicine, Shandong University, Jinan, Shandong, China; 2 Division of Gastroenterology, Department of Internal Medicine, University of Michigan Health System, Ann Arbor, Michigan, United States of America; 3 Unit for Laboratory Animal Medicine, University of Michigan Medical School, Ann Arbor, Michigan, United States of America; 4 Department of Surgery, University of Michigan Health System, Ann Arbor, Michigan, United States of America; Veterans Affairs Medical Center (111D), United States of America

## Abstract

We have shown that *Helicobacter pylori* induces tolerogenic programming of dendritic cells and inhibits the host immune response. Toll-like receptors (TLRs) represent a class of transmembrane pattern recognition receptors essential for microbial recognition and control of the innate immune response. In this study, we examined the role of TLRs in mediating *H. pylori* tolerogenic programming of dendritic cells and their impact on anti–*H. pylori* immunity using C57BL/6 wild-type and TLR2-knockout (TLR2KO) mice. We analyzed the response of TLR2KO bone marrow-derived dendritic cells (BMDCs) to *H. pylori* SS1 stimulation and the outcome of chronic *H. pylori* infection in TLR2KO mice. We showed that *H. pylori*–stimulated BMDCs upregulated the expression of TLR2, but not TLR4, TLR5, or TLR9. *H. pylori*-stimulated BMDCs from TLRKO mice induced lower Treg and Th17 responses, but a higher IFN-γ response compared to *H. pylori*-stimulated BMDCs from wild-type mice. In vivo analyses following an *H. pylori* infection of 2 months duration showed a lower degree of gastric *H. pylori* colonization in TLR2KO mice and more severe gastric immunopathology compared to WT mice. The gastric mucosa of the infected TLR2KO mice showed a lower mRNA expression of Foxp3, IL-10, and IL-17A, but higher expression of IFN-γ compared to the gastric mRNA expression in infected wild-type mice. Moreover, the *H. pylori*–specific Th1 response was higher and the Treg and Th17 responses were lower in the spleens of infected TLR2KO mice compared to infected WT mice. Our data indicate that *H. pylori* mediates immune tolerance through TLR2-derived signals and inhibits Th1 immunity, thus evading host defense. TLR2 may be an important target in the modulation of the host response to *H. pylori*.

## Introduction

*Helicobacter pylori* is a highly adaptive gram-negative bacterium that colonizes the human stomach and thus contributes to diseases such as gastric ulcers and cancers. Infected individuals generate a vigorous systemic and mucosal humoral response [1] but fail to eradicate the offending organism and thus are susceptible to mucosal damage [2]. Emerging evidence suggests that failure to eradicate *H. pylori* may be attributed to *H. pylori*’s ability to induce a regulatory T cell (Treg) response against helper T cell immunity. *H. pylori*–specific CD4^+^CD25^+^ Tregs have been shown to suppress memory T cell responses to *H. pylori* in infected individuals [3,4]. Expression of the Treg marker Foxp3 mRNA is higher in the gastric tissue of *H pylori*–infected persons compared with uninfected controls [5-7]. Moreover, Harris et al. [8] reported an inverse correlation between gastric Foxp3 expression and gastric pathology in *H pylori*–infected children compared to adults. We have shown that *H. pylori* can induce tolerogenic programing of dendritic cells (DCs) and inhibit the host immune response [9,10] and other researchers have shown that DCs are involved in the host response to *H. pylori* infection [11].

Pattern recognition receptors such as the Toll-like receptors (TLRs) on antigen-presenting cells (e.g., DCs and macrophages) are important in the modulation of host immune responses. To date, 10 and 12 functional TLRs have been identified in humans and mice, respectively [12]. Each TLR recognizes distinct pathogen-associated molecular patterns (PAMPs) derived from bacteria, viruses, mycobacteria, fungi, and parasites. TLR2, which is expressed on the surfaces of intestinal and gastric epithelial cells [13], recognizes lipopeptides from bacteria, peptidoglycans and lipoteichoic acid from gram-positive bacteria, and lipoarabinomannan from mycobacteria. Smith et al. suggested that TLR2 plays a role in the recognition of *H. pylori* and the subsequent induction of intracellular signaling cascades that activate inflammation [14]. In general, stimulation of the TLRs on DCs results in upregulation of costimulatory molecules, secretion of cytokines, and enhanced uptake and presentation of antigens [15].

Research indicates that TLR2 may direct tolerogenic responses. In a mouse model of *Candida albicans* infection, TLR2-derived signals induced immunosuppression and an increase in IL-10 production and Treg cell survival [16]. Others studies have shown that TLR2 enhances the expansion and function of CD4^+^CD25^+^ Treg cells through Foxp3 expression [17,18]. Little is known about the role of TLR2 in DC activation in response to *H. pylori*.

In this study, we examined the role of TLRs in mediating *H. pylori* tolerogenic programming of DCs and their impact on anti–*H. pylori* immunity. We showed in vitro that *H. pylori*–stimulated BMDCs upregulated the expression of TLR2, but not TLR4, TLR5, or TLR9. *H. pylori*–stimulated BMDCs from TLR2 knockout (TLR2KO) mice induced lower Treg and Th17 responses, but a higher IFN-γ response compared to *H. pylori*–stimulated BMDCs from wild-type (WT) mice. In vivo analyses after an *H. pylori* infection of 2 months duration showed lower degree of gastric *H. pylori* colonization in TLR2KO mice and more severe gastritis compared to WT mice. The gastric mucosa of the infected TLR2KO mice showed lower Treg and Th17 responses, but a higher Th1 response compared to infected WT mice. Moreover, a higher *H. pylori*–specific Th1 response and lower Treg and Th17 responses were measured in the spleens of infected TLR2KO mice. Our data indicate TLR2 may be an important target in the modulation of the host response to *H. pylori*.

## Materials and Methods

### Mice

Specific pathogen–free female C57BL/6 mice aged 6–8 weeks and TLR2-deficient mice were purchased from Jackson Laboratory (Bar Harbor, ME). All animals were housed in the animal maintenance facility at the University of Michigan Health System. This research was undertaken with the approval of the University Committee on Use and Care of Animals at the University of Michigan. Mouse genotypes were confirmed by PCR with tail genomic DNA.

### Media and cytokines

For all experiments, complete medium consisted of RPMI-1640 (Sigma-Aldrich, St. Louis, MO) with 9% heat-inactivated fetal calf serum (BioExpress, Kaysville, UT), 2 mM added glutamine (4 mM total), and 100 U/mL penicillin–streptomycin. The recombinant mouse cytokines (GM-CSF, IL-4, and Flt3L, R&D Systems, Minneapolis, MN) were diluted in complete medium. After the cell harvest on day 6, only GM-CSF was included in the complete medium for the duration of the experiment, i.e., through the stimulation, rest, and re-stimulation periods. TLR2 ligand, Pam3Cys (EMC Microcollections GmbH, Tübingen, Germany) was used for in vitro experiments.

### Bacterial strains and culture condition

*H. pylori* was grown on *Campylobacter* -selective agar (BD Diagnostics, Bedford, MA) for 3–5 days in a humidified microaerophilic chamber at 37°C (BBL, Gas System, with CampyPak Plus packs, BD Biosciences, San Jose, CA). All experiments were performed using *H. pylori* strain SS1. To prepare the bacterial sonicate, bacteria were diluted in phosphate-buffered saline (PBS, Invitrogen, Frederick, MD) to a concentration of 1 × 10^9^/mL and subjected to repeated sonication in an ultrasonic bath. *H. pylori* were identified by colony morphology and through positive biochemical tests for ureases, catalase, and oxidase. Infections were performed by oral gavage with 10^9^ bacteria suspended in 100 μL of Brucella broth. Protein levels were assayed using a BSA standard (Bio-Rad Laboratories, Hercules, CA), and overall protein concentration was used as representative of proportional amounts of all bacterial components. For further experiments, *H. pylori* was prepared in 0.9% saline solution at a concentration of 1 × 10^9^ bacteria/mL, which was measured by optical density determination at 600 nm and adjusted to a final absorbance of 0.75.

### Generation and stimulation of BMDCs

BMDCs were generated as previously described [19]. Briefly, erythrocyte-depleted murine bone marrow cells were cultured in complete medium with cytokines. On day 6, DCs were harvested by vigorous pipetting and enriched by gradient centrifugation (OptiPrep^TM^, Sigma-Aldrich). DCs were collected by gentle aspiration at the low-density interface, washed twice, and cultured in complete medium with mouse GM-CSF (10 ng/mL) and IL-4 (10 ng/mL). During optimization of the BMDC protocol, alternative culture conditions included supplementation with 10 ng/mL mouse GM-CSF or Flt3L alone.

BMDCs (10^6^ cells/mL) were treated with either live *H. pylori* (10^8^ CFU/mL) or *H. pylori* sonicate (10 μg/mL). The cells were then washed 3 times with PBS, re-plated with complete medium, and allowed to rest overnight. For the coculture of BMDCs with syngeneic splenocytes in a mixed leukocyte reaction, the spleens were harvested, crushed, and filtered through 100 μm filters and the red blood cells were lysed with ammonium-chloride-potassium (ACK) lysing solution. The mixed leukocyte reaction was performed with a splenocyte-to-BMDC ratio of 10 to 1. Supernatants were collected for cytokine analysis at 72 h.

### Animal studies

C57BL/6 and TLR2KO mice were orally gavaged with *H. pylori* SS1 (3 times over 1 week). After 2 months, the mice were analyzed. The stomach was cut along the greater curvature and removed. Strips (2 mm) composed of fundus and antrum were embedded in Tissue-Tek optimum cutting temperature (OCT) compound (Sakura Finetek, Torrance, CA) and immersed in liquid nitrogen. Paraffin sections were also prepared for H&E staining. Meanwhile, splenocytes from C57BL/6 and TLR2KO mice were cocultured for 18 h with BMDCs infected with *H. pylori*. The splenocyte-to-BMDC ratio was 10 to 1. After 72 h, splenocytes were collected and the percentages of IFN-γ, IL-17A, and Foxp3 were measured by flow cytometry (i.e., FACS, fluorescence-activated cell sorting).

### Measurement of cytokine production by BMDCs and splenocytes

The supernatant from the coculture of BMDCs and splenocytes was collected and the concentrations of IL-12p70, IL-23p19, IL-6, TNF-α, IFN-γ, IL-10, and IL-17A were measured using Quantikine ELISA Kits (eBioscience, San Diego, CA, or BD Biosciences, San Jose, CA).

### Determination of the expression of intracellular markers on splenocytes and surface markers on BMDCs by flow cytometry

Splenocytes were labelled with fluorescence-conjugated antibodies to Foxp3, IFN-γ, and IL-17A (eBioscience). Stimulated BMDCs were dual-labelled with fluorescence-conjugated antibodies to CD11c and TLR2 or TLR4. For cytokine profiles, the cells were stimulated, stained, and analyzed, as previously published [20], with a Coulter XL Flow Cytometer (Beckman Coulter, Miami, FL).

### Histological analysis

After *H. pylori* infection of 2 months duration, the stomachs of WT and TLR2KO mice were removed and measured. Two adjacent full-thickness longitudinal strips were removed from the greater curvature and fixed in formalin for histological analysis. The specimens were scored separately for the presence or absence of each of the following histologic criteria: neutrophil infiltration (polymorphonuclear neutrophils), gastritis, and epithelial metaplasia. The results were reported as the percentage of fields affected each slide [21].

### Immunohistochemistry

Paraffin sections (8μm) were de-waxed in xylene twice, incubated in 100%, 95%, and 70% ethanol for 1 minute each, and hydrated in water for 5 minutes. Antigen retrieval was performed in 10mM sodium citrate, pH6, in the microwave for 5 minutes after boiling temperature was reached, and then incubated for 20 minutes at room temperature. For HRP-based detection, the slides were incubated in 0.09% hydrogen peroxide in methanol solution for 20 minutes at room temperature in the dark. The sections were equilibrated in 0.01% Triton X-100/PBS (PBST) (Sigma, St Louis, MO) for 5 minutes at room temperature. The tissue sections were then blocked in 20% donkey serum (Cat# 017-000-121, Jackson ImmunoResearch, West Grove, PA) plus 1% BSA (Fisher Scientific, Houston, TX) for 20 minutes, followed by primary antibody incubation overnight at 4°C. The primary antibodies used were: rabbit monoclonal against Ki67 (1:200, Cat# RM-9106-S1, Thermo Scientific, Fisher, Houston, TX), rabbit monoclonal against cleaved-Caspase 3 (1:50, Cat# 9664, Cell signaling, Boston, MA), and rabbit polyclonal against myeloperoxidase (MPO, 1:50, Cat# ab9535, Abcam, Cambridge, MA). The next day, the slides were washed three times in PBST. For HRP-based detection, the staining was developed using the biotin-streptavidin (ABC) IHC detection kit (Abcam), counterstained with Permount (Fisher Scientific), and counterstained with hematoxylin. For Fluorescent detection, the slides were incubated with 1:500 donkey anti-rabbit Alexa Fluor-conjugated secondary antibody (Molecular Probes, Invitrogen, Carlsbad, CA), and washed twice in PBST. The sections were stained mounted with ProLong Gold antifade reagent plus DAPI (Invitrogen), and visualized using an Olympus Fluoview scanning confocal microscope (Olympus, Center Valley, PA). Morphometric analyses were performed by observing well-oriented gastric glands from the fundus under a 20× objective lens (200× total magnification). The number of immunoreactive cells per 20x field view or per gland was measured and expressed as mean ± S.E.M. of the total number of fields examined.

### Extraction of RNA, reverse transcription, and quantitative real-time polymerase chain reaction (RT-PCR)

Total RNA from WT and TLR2-deficient mouse stomachs was extracted using TRIzol reagent and reverse transcribed to cDNA using the PrimeScript RT reagent kit (Takara, Otsu, Shiga, Japan). cDNA was amplified with SYBR Green Master (Roche, Basel, Switzerland) in a 7300 Real-time PCR System (Applied Biosystems, Foster City, CA). Primers specific for GAPDH (glyceraldehyde 3-phosphate dehydrogenase), IL-6, Foxp3, IFN-γ, IL-10, IL-17A, *H. pylori*/UraEa, and *H. pylori* 16s are shown in Table 1. Real-time PCR was run in triplicate in a volume of 20 µL containing 10 µL of SYBR Green PCR Master, 0.2 µL of each primer, and 2 µL cDNA. Reaction conditions were as described for the SYBR Green kit, and the cycling protocol was as follows: 95°C for 10 min, 40 cycles of 95°C for 15 s, 60°C for 30 s. The housekeeping gene GAPDH was used as an internal control. Finally, quantitation of relative differences in expression was calculated using the comparative 2^-ΔΔ^CT method [22]. Mouse TNF-α primers are forward 5’-ggctttccgaattcactggag-3’ and reverse 5’-ccccgcccttccaaataaa-3’.

**Table 1:** Primers and annealing temperatures used for the amplification of each gene.

Gene	Primer (5′- 3′)	Annealing temperature (°C)		Product size (base pairs)
GAPDH	F:5′-TCAAGAAGGTGGGTGAAGCAGG-3′	60		350
	R:5′-TATTATGGGGGTCTGGGATGG-3′			
IL-6	F:5′-CTACCCCAATTTCCAATGCT-3′	58		187
	R:5′-ACCACAGTGAGGAATGTCCA-3′			
IL-17A	F:5′-GCTCCAGAAGGCCCTCAGA-3′	57		142
	R:5′-AGCTTTCCCTCCGCATTGA-3′			
IL-10	F:5′-AGTGGAGCAGGTGAAGAGTG-3′	58	250	
	R:5′-TTCGGAGAGAGGTACAAACG-3′			
IFN-γ	F:5′-TCAAGTGGCATAGATGTGGAAGAA-3′	60	92	
	R:5′-TGGCTCTGCAGGATTTTCATG-3′			
Foxp3	F:5′-TCTCCAGGTTGCTCAAAGTC-3′	58	203	
	R:5′-GCAGAAGTTGCTGCTTTAGG-3′			
HP-Ura	F:5′-AAACCGGATGATGTGATG-3′	55	350	
	R:5′-GGTATGCACGGTTACGAG-3′			
HP 16s	F:5′-CAAGTCATGGCCCTTAC-3′	58	161	
	R:5′-TTGCGATTACTAGCGATTCC-3′			

### Statistical analysis

The results were evaluated using an unpaired Student *t* test (means ± SEM). Statistical tests were performed using GraphPad Prism (GraphPad Software, La Jolla, CA). Significant values were indicated as follows: *P < 0.05, **P < 0.01 and ***P < 0.001.

## Results

### Increased CD11c^HI^MHCII^HI^ DCs in *H. pylori*–infected mouse stomach and the expression of TLRs by *H. pylori*–stimulated BMDCs

**Figure 1:**
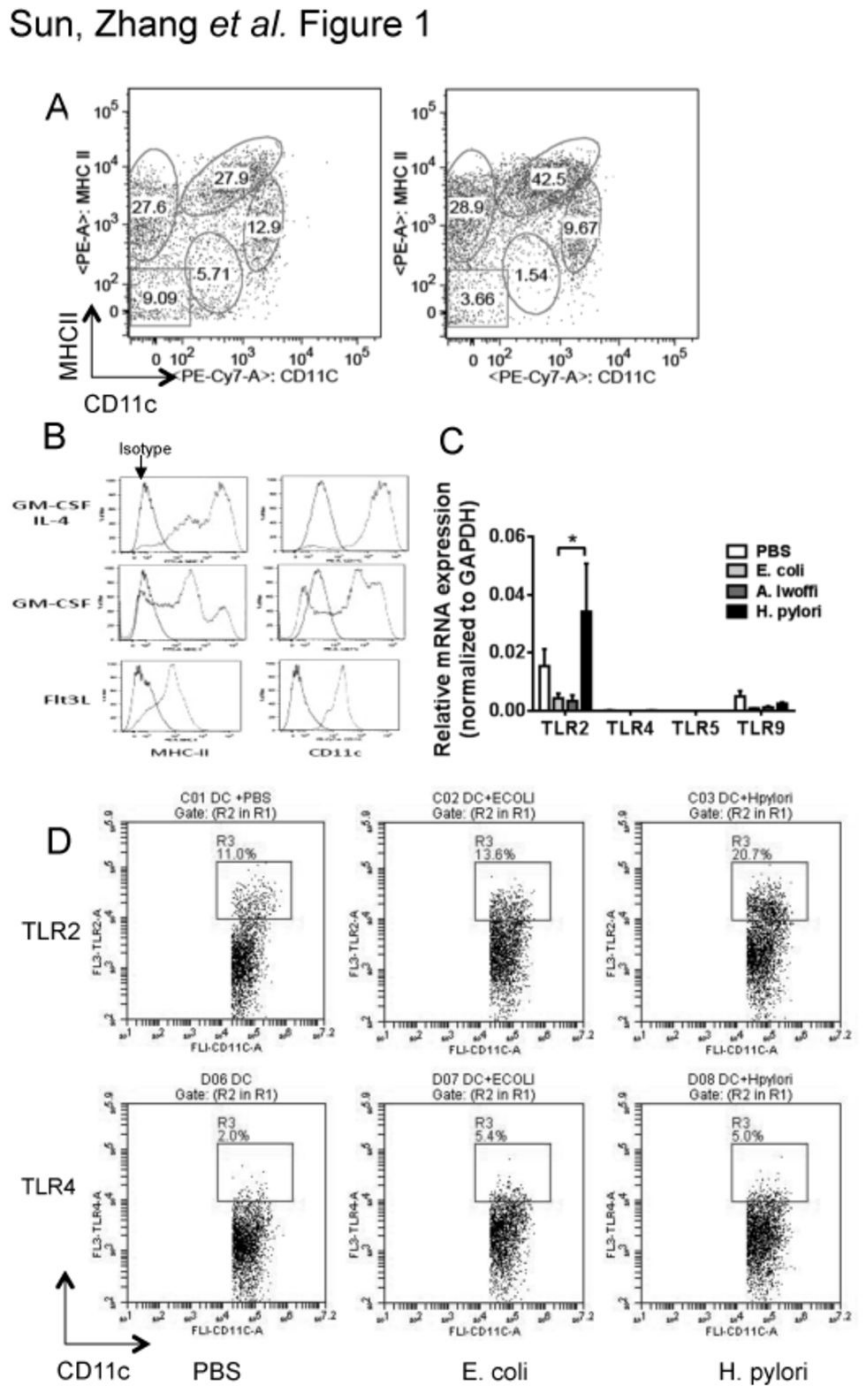
Increased CD11c^HI^MHCII^HI^ DCs in *H. pylori*–infected mouse stomach and the expression of TLRs by *H. pylori*-stimulated BMDCs. **a**, Single cell prep of stomach from uninfected C57BL/6 mice or from mice infected with *H*. *pylori* for 24 h were dual-labeled with phycoerythrin (PE)-conjugated anti-mouse MHC II and PE and a cyanine dye (Cy7)-conjugated anti-mouse CD11c antibodies and analyzed by FACS. **b**, BMDCs from C57BL/6 mice were cultured in culture medium supplemented with GM-CSF plus IL-4, GM-CSF only, or Flt3L.The expression of MHC II and CD11c was analyzed by flow cytometry on day 6. Background staining was assessed using isotype controls. BMDCs in the GM-CSF plus IL-4 group had higher levels of MHC II and CD11c than cells in the GM-CSF group or Flt3L group. **c**, BMDCs were pulsed with PBS, live *Escherichia* *coli* (EC), live *Acinetobacter* *lwoffii* (AL), or live *H*. *pylori* (HP) for 18 h (multiplicity of infection, 10:1). The mRNA expressions of TLR 2, TLR4, TLR5, and TLR9 were measured by quantitative PCR. Data shown were obtained from three independent experiments. **d**, BMDCs were pulsed with PBS, live *Escherichia* *coli* (EC), or live *H*. *pylori* (HP) for 18 h (multiplicity of infection, 10:1). BMDCs were dual-labeled with fluorescence-conjugated CD11c and TLR2 or TLR4 and analyzed by flow cytometry. Representative dot plots from three separate experiments are shown.

To determine the role of DCs during *H. pylori* infection, lamina propria (LP) cells isolated from the stomachs of *H. pylori*–infected mice were analyzed using FACS. We identified five subsets of LP cells in normal mouse stomach based on their CD11c and MHC class II status. After *H. pylori* infection, the percentage of the CD11c^HI^MHCII^HI^ gastric DC subset increased significantly compared to its presence in uninfected stomach (Figure 1a). Attempts to obtain enough CD11c^HI^MHCII^HI^ DCs for further in vitro characterization were not possible due to low cell numbers. Therefore, we used three different methods of deriving DCs from bone marrow and found that supplementation of the culture medium with GM-CSF/IL-4 produced the highest yield of the CD11c^HI^MHCII^HI^ DCs compared to supplementation with GM-CSF or FLT-3L alone (Figure 1b). To determine which TLRs on BMDCs are significantly upregulated, we measured mRNA expression of TLR2, TLR4, TLR5, and TLR9 and found a significant increase in TLR2 expression by *H. pylori*–stimulated BMDCs but not by *Escherichia coli*- or *Acinetobacter* *lwoffii* –stimulated BMDCs (Figure 1c). Next, we measured the protein levels of TLR2 and TLR4 on BMDCs and found a higher level of TLR2, but not TLR4, on *H. pylori*–stimulated BMDCs compared to *Escherichia coli*–stimulated BMDCs (Figure 1d). We previously showed that *H. pylori*–stimulated BMDCs induced a significantly higher level of Treg response compared to *E. coli*- or *A* *. lwoffii* *–stimulated* BMDCs [9]. These results provided the rationale for the examination of the role of TLR2 in directing the tolerogenic programming of *H. pylori*–stimulated dendritic cells.

### *H. pylori*–stimulated BMDCs from TLR2KO mice produced lower levels of proinflammatory cytokines

**Figure 2:**
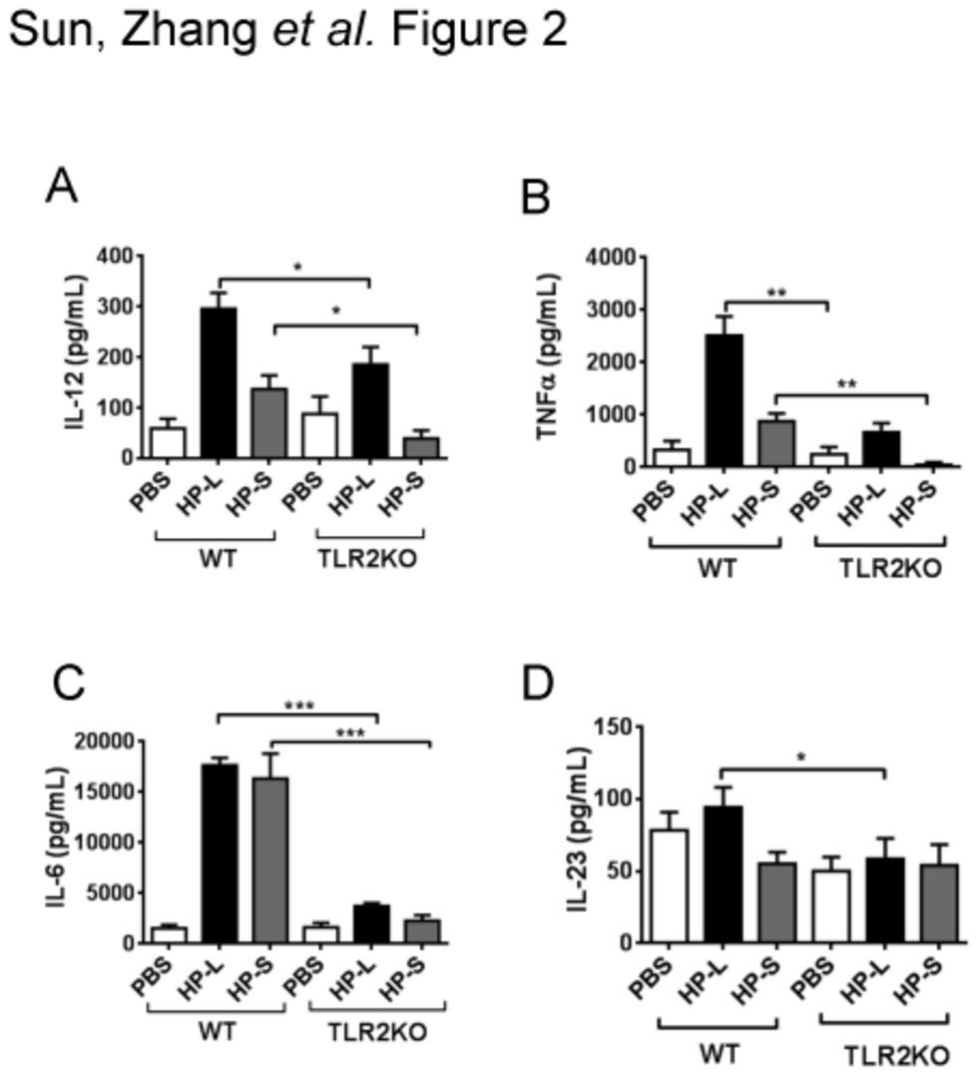
*H. pylori*-stimulated BMDCs from TLR2KO mice produced lower levels of proinflammatory cytokines. BMDCs derived from C57BL/6 mice (wild type, WT) and TLR2KO mice were stimulated with a blank control, live *H*. *pylori* (HP-L), and *H*. *pylori* sonicate (HP-S). At 18 h post infection, the supernatants were collected from medium alone and HP-L- and HP-S–treated BMDCs. The concentrations of IL-12p70 (**a**), TNF-α (**b**), IL-6 (**c**), and IL-23p19 (**d**) in supernatant were determined by enzyme-linked immune absorbent assay (ELISA). Data are representative of the results of three independent experiments. Data are presented as means ± SEM of 3 mice in vitro. ^*^ *P* < 0.05, ^**^ *P* < 0.01, and ^***^ *P* < 0.01 compared with WT mice.

To determine the role of TLR2 in mediating the response of BMDCs to *H. pylori*, the production of proinflammatory cytokines by *H. pylori*–stimulated BMDCs was compared in WT and TLR2KO mice. As shown in Figure 2, TLR2KO BMDCs stimulated by live *H. pylori* or *H. pylori* sonicate produced lower levels of proinflammatory cytokines important for Th1 (e.g., IL-12 and TNF- α) and Th17 (e.g., IL-6 and IL-23) compared to WT BMDCs. Baseline cytokine production was the same in both WT and TLR2KO BMDCs. In both groups, BMDC cytokine production was higher with stimulation by live *H. pylori* than by *H. pylori* sonicate. These data suggest a role for TLR2 in the modulation of the response of BMDCs to *H. pylori*, consistent with a previous report by Uno et al. [23].

### *H. pylori*–stimulated TLR2KO BMDCs induced a skewed Th1 response

**Figure 3:**
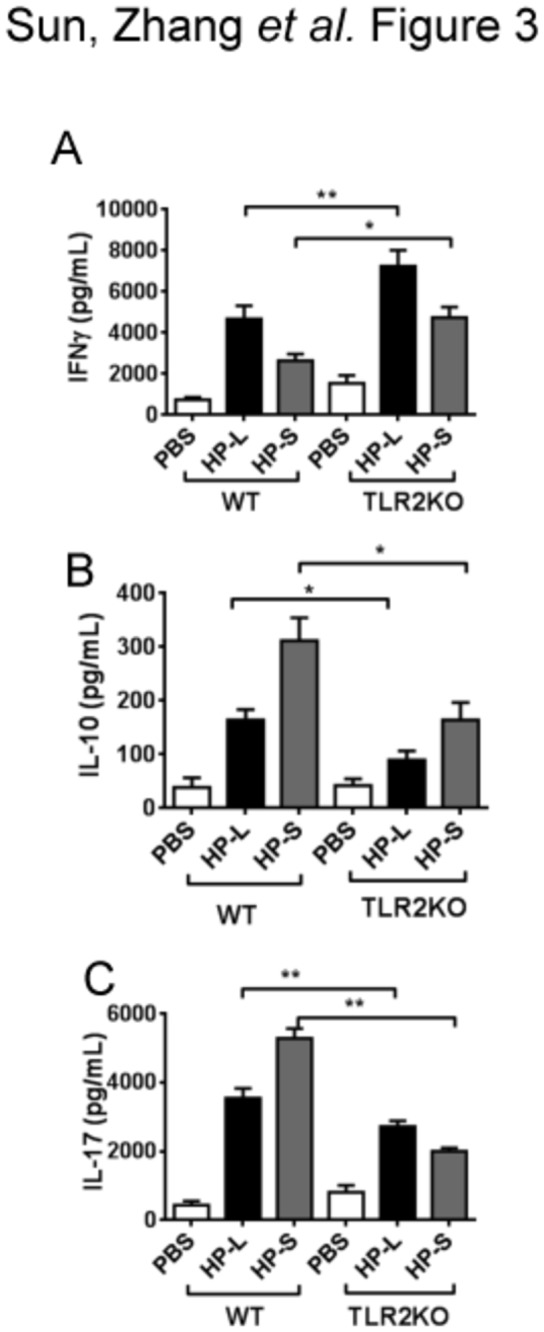
*H. pylori*–stimulated TLR2KO BMDCs induced a skewed Th1 response. BMDCs from WT mice and TLR2KO mice were stimulated with a blank control, live *H*. *pylori* (HP-L), or *H*. *pylori* sonicate (HP-S). DCs (1 × 10^5^ cells/well) were prepared 18 h after *H*. *pylori* infection and cocultured with naive syngeneic splenocytes (1 × 10^6^ cells/well) for 72 h at a splenocyte-to-DC ratio (10:1). Supernatants were collected and protein expression was measured by ELISA. The secretion of IFN-γ (**a**) was higher, whereas the secretion of IL-10 (**b**) and IL-17A (**c**) was lower in TLR2KO mice than in WT mice (^*^ *P* < 0.05 and ^**^ *P* < 0.01 compared with WT mice).

To determine the functional significance of TLR2 in the ability of DCs to induce helper T cell responses, we performed a mixed leukocyte reaction by coculturing *H. pylori*–stimulated BMDCs from WT and TLR2KO mice with WT splenocytes. Compared to *H. pylori*–stimulated WT BMDCs, *H. pylori*–stimulated TLR2KO BMDCs induced a higher splenocyte production of IFN-γ (Th1 response) and lower production of IL-17 (Th17 response) and IL-10 (Treg response) (Figure 3). These findings indicate that TLR2 signaling during BMDC recognition of *H. pylori* may skew differentiation of naive T cells toward Th17 and Treg responses and away from a Th1 response. To assess whether this pattern of T cell differentiation induced by *H. pylori*–stimulated BMDCs is unique to *H. pylori*, a synthetic TLR2 ligand, Pam3Cys, was used to stimulate BMDCs. The helper T cell responses induced by Pam3Cys- or *H. pylori*–stimulated BMDCs were compared. As shown in Figure S1, Pam3Cys–stimulated BMDCs induced a higher level IL-17 (Th17) and IFN-γ (Th1), but a lower level of Foxp3 (Treg) than *H. pylori*–stimulated BMDCs indicating that *H. pylori* TLR2 ligand uniquely skews Th17 and Treg differentiation.

### Increased gastritis and decreased *H. pylori* colonization in TLR2KO mice

**Figure 4:**
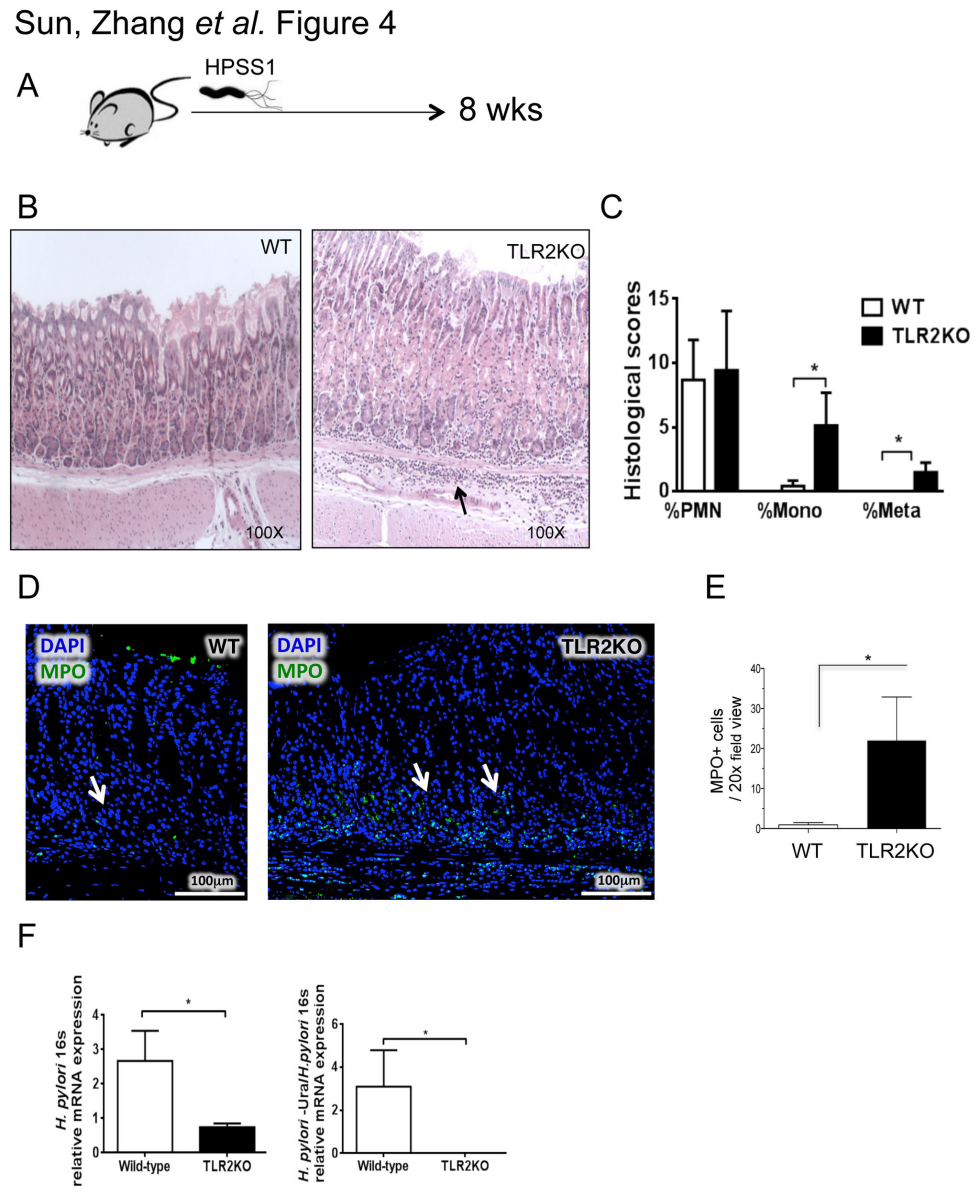
Increased gastritis and decreased *H. pylori* colonization in TLR2KO mice. C57BL/6 mice (n = 10 per group) were orally challenged with *H*. *pylori* SS1 (10^9^ CFU/mL) 3 times in 1 week starting on day 0. Mice were analyzed after 8 weeks. **a**, Schematic representation of the animal study. **b**, Micrographs of gastric histology (arrow pointing to area of inflammatory infiltrate). **c**, Gastric score was determined in a blinded fashion. **d** and **e**, Neutrophilic infiltration is increased in TLR2KO mice. Confocal immunofluorescent detection of MPO (green) and DAPI (blue) in the gastric fundus of *H*. *pylori*-infected WT versus TLR2KO mice. Morphometric analysis of MPO+ cells was measured and expressed as mean ± S.E.M. per 20x filed view. **f**, *H*. *pylori* colonization was determined by quantitative PCR of H. pylori 16S and *H*. *pylori*–Ura/*H*. *pylori* 16S mRNA. Results are showin as means ± S.E.M.. ^*^ *P* < 0.05 and ^**^ *P* < 0.01 compared with WT mice.

**Figure 5:**
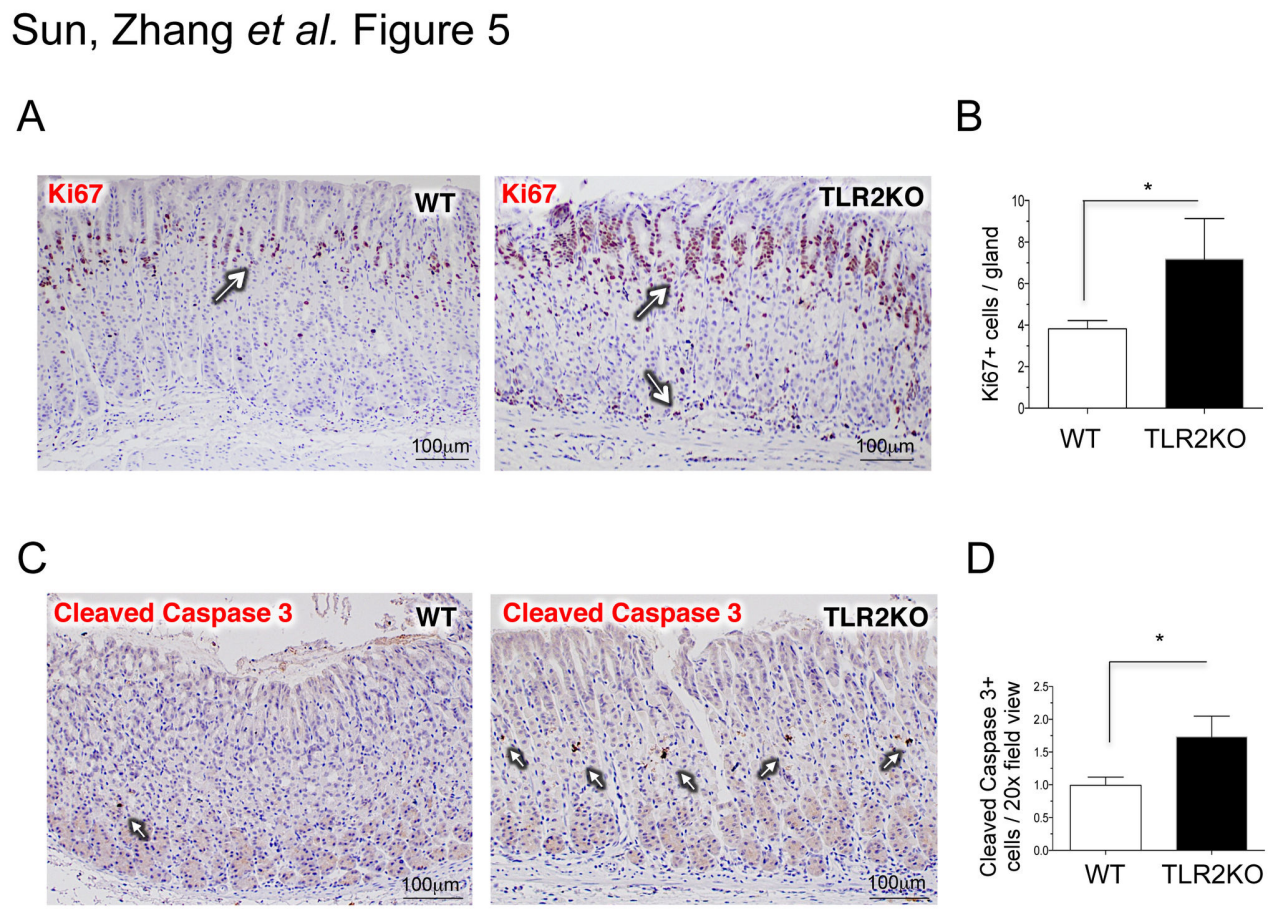
Increased cellular turnover in *H. pylori*–infected TLR2KO mice. C57BL/6 mice (n = 4-5 per group) were orally challenged with *H*. *pylori* SS1 (10^9^ CFU/mL) 3 times in 1 week starting on day 0. Mice were analyzed after 8 weeks. Immunohistological staining of gastric sections was performed. **a** and **c**, Cellular proliferation was measured by Ki67 and apoptosis was measured by cleaved-caspase 3. **b** and **d**, Morphometric analysis was performed by counting the number of Ki67+ cells or cleaved-caspase 3+ cells expressed as mean ± S.E.M. per gland or per 20x filed view. ^*^ *P* < 0.05 compared with WT mice. Arrows are pointing to areas with positive staining.

To examine the significance of TLR2 signaling during chronic *H. pylori* infection, WT and TLR2KO mice were infected chronically for 2 months with *H. pylori* SS1 (Figure 4a). Histological indices (i.e., percentages of polymorphonuclear neutrophil and monocyte infiltration and metaplasia) and *H. pylori* colonization were measured. A higher degree of gastric inflammation with increased neutrophilic infiltration was measured in TLR2KO mice compared to WT mice (H&E, Figure 4b and 4c; MPO staining, Figure 4d and 4e). A significantly lower degree of *H. pylori* colonization was measured by quantitative PCR (Figure 4f). Additional histopathological analyses indicated increased cellular turnover (Ki67-proliferation, Figure 5a and 5b; cleaved-caspase 3-apoptosis, Figure 5c and 5d). These findings indicate that *H. pylori* signaling through TLR2 affords it a survival advantage.

### Reduced Th17/Treg and increased Th1 responses in *H. pylori*–infected TLR2KO mice

**Figure 6:**
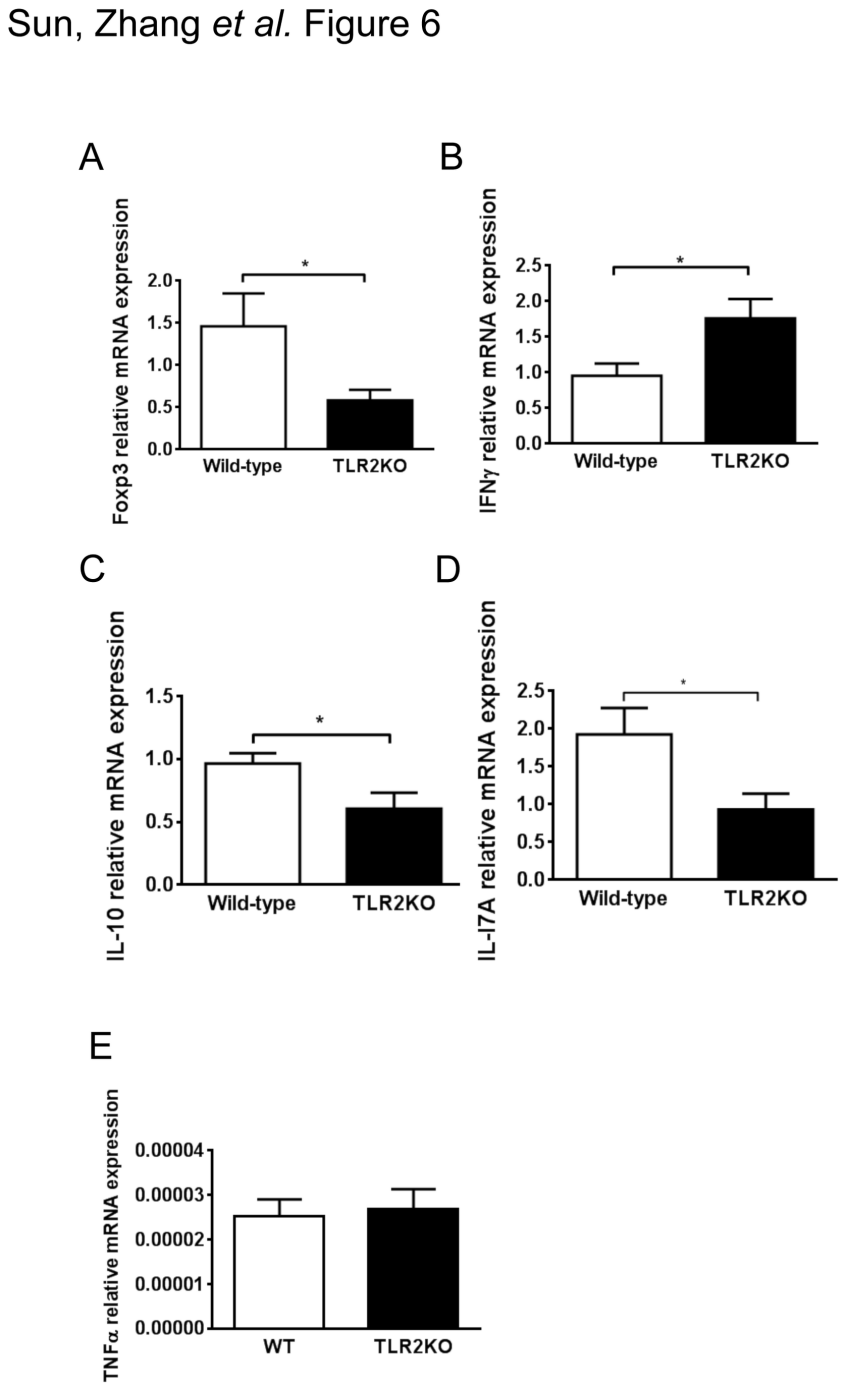
Reduced Th17/Treg and increased Th1 responses in *H. pylori*–infected TLR2KO mice. After an *H*. *pylori* (10^9^ CFU/mL) infection of 2 months duration, stomachs from WT mice and TLR2-deficient mice were removed. mRNA expression of Foxp3 (**a**), IFN-γ (**b**), IL-10 (**c**), IL-17A (**d**), TNF-α (**e**) and the housekeeping gene GAPDH were measured by quantitative PCR (n = 10 mice per group; ^*^ *P* < 0.05).

Based on our in vitro observation that TLR2 deficiency in BMDCs resulted in skewing toward a Th1 response, we hypothesized that a more robust Th1 immune response in TLR2KO mice vs WT mice will lead to decreased survival of the bacteria. Thus, we measured the mRNA expression of helper T cell cytokines (Foxp3, IFN-γ, IL-10, IL-17A) in the stomach. As shown in Figure 6a-d, we measured a reduced mRNA expression of Foxp3/IL-10 and IL-17A and an increased expression of IFN-γ in TLR2KO mice compared to WT mice. No significant difference in TNF-α was measured (Figure 6e) suggesting TLR2 does not significantly alter the induction of gastric TNF-α expression in vivo during chronic *H. pylori* infection. These results indicate that, in vivo, TLR2 signaling supports the adaptive Treg/Th17 response during *H. pylori* infection and inhibits Th1 immunity.

### Enhanced *H. pylori*–specific Th1 response in chronically infected TLR2KO mice

**Figure 7:**
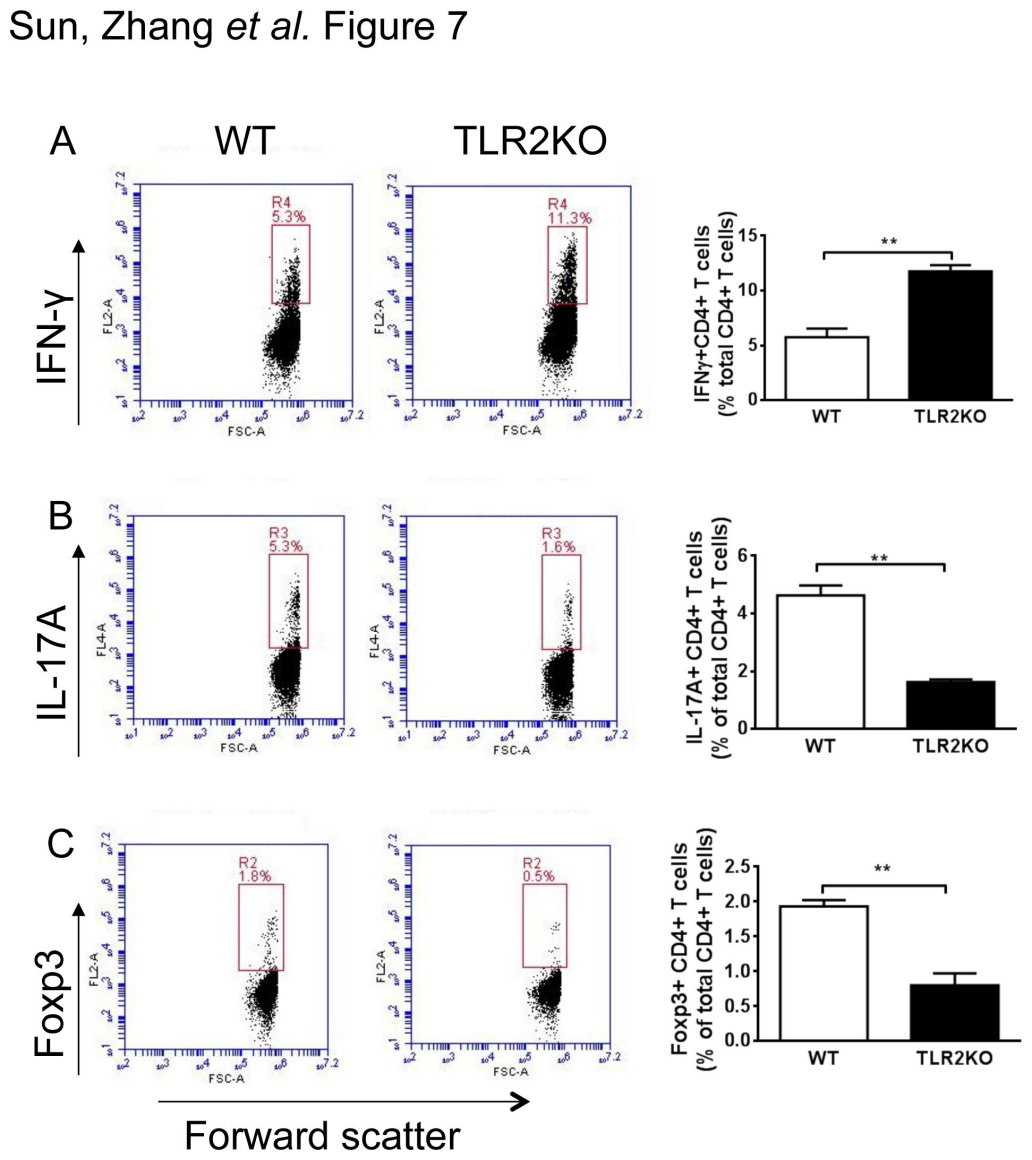
Enhanced *H. pylori*–specific Th1 response in chronically infected TLR2KO mice. BMDCs from WT mice were cultured. On day 6, 1 × 10^6^ cells/mL of BMDCs were treated with *H*. *pylori* (H. *pylori* to DC ratio was 10 to 1). DCs were prepared 18 h after infection with *H*. *pylori* and cocultured with splenocytes from WT mice or TLR2KO mice after *H*. *pylori* infection of 2 months duration. During the 72 h coculture, the splenocyte-to-DC ratio was 10 to 1. The percentages of IFN-γ, IL-17A, and Foxp3 from splenocytes were determined using an intracellular cytokine staining assay. Representative flow plots demonstrate the increase of IFN-γ and the decrease of IL-17A and Foxp3 in TLR2KO splenocytes compared to WT splenocytes with gating held constant. At the same time, results are expressed as the mean of three sample replicates. A higher population of IFN-γ–positive cells are seen in TLR2KO splenocytes stimulated by *H*. *pylori*. In contrast, a lower population of IL-17A and Foxp3 cells are seen in TLR2KO splenocytes after *H*. *pylori* infection. Data represent means ± SEM, n = 5 in duplicate.

To determine if the helper T cell cytokines measured in the *H. pylori*–infected stomach are specific to *H. pylori*, splenocytes from these mice were analyzed for their ability to produce helper T cell cytokines in the presence of *H. pylori*. Similar to findings in the stomach and in vitro BMDCs, *H. pylori*–infected TLR2KO mice had an increased *H. pylori*–specific Th1 response (IFN-γ) but dampened Th17 (IL-17) and Treg (Foxp3) responses compared to the responses in WT mice (Figure 7). Our data indicate that signaling through TLR2 during *H. pylori* infection enhances the induction of Treg and Th17 and inhibits the induction of Th1.

## Discussion

We and others have reported that *H. pylori* immune escape is mediated by DC-induced Treg skewing [9,24]. In the current study, we show that *H. pylori* infection results in an increase in the percentage of the CD11c^HI^MHCII^HI^ DCs. Attempts to isolate these cells for functional studies were unsuccessful due to low yield and poor cell survival in vitro (data not shown). Using CD11c^HI^MHCII^HI^ BMDCs, we found that *H. pylori* uniquely upregulates the expression of TLR2 but not TLR4, 5, or 9. To determine the significance of TLR2 upregulation by *H. pylori* stimulation, we analyzed the response of wild-type and TLR2KO BMDCs to *H. pylori*. TLR2KO BMDCs produced lower levels of proinflammatory cytokines and induced lower Th17 and Treg responses, but a higher Th1 response. In vivo, TLR2KO mice are poorly colonized by *H. pylori* and histological analyses revealed more severe gastric immunopathology (e.g., higher percentages of gastric mucosa with monocytes and metaplasia, increased MPO staining and cellular turnover). Similar to our in vitro observation, *H. pylori*–infected TLR2KO mice exhibited a Th1 skewed cellular response in both the stomach and the spleen. Our data indicate that *H. pylori* mediates immune tolerance through TLR2-derived signals and inhibits Th1 immunity, thus evading host defense.

In this study, we showed that *H. pylori*–stimulated BMDCs only upregulated the expression of TLR2. The mechanism for TLR2 upregulation by *H. pylori* is unclear, however, it is known that there is a positive feedback regulation of TLR expression [25]. Because *H. pylori* activates host immune response predominantly through TLR2 [26], this would result in an upregulation of TLR2 and not other TLRs. Mandell L et al. have shown that cag pathogenicity island genes may modulate TLR2 activity of *H. pylori* [26] which may be another potential mechanism of TLR2 upregulation by *H. pylori*. More studies are needed to understand the exact mechanisms involved.

TLRs are involved in the identification of PAMPS during *H. pylori* infection [27,28]. Many immune responses in vivo, such as the secretion of proinflammatory cytokines and chemokines, depend almost exclusively on TLR2 [29]. Rad et al. showed that TLR2 was the major surface receptor mediating the cytokine response of BMDCs to *H pylori* [27]. Our study supports this finding and further elucidates the role of TLR2 in DC priming of adaptive helper T cell responses against *H. pylori* and *H. pylori* survival. We found TLR2 to be essential for *H. pylori*–induced Treg and Th17 responses and further, that TLR2 deficiency results in an enhanced Th1 response. The importance of Th1 immunity against *H. pylori* is well established [30-32]. Thus, the ability of *H. pylori* to evade host immunity and colonize the stomach requires TLR2-dependent Treg induction and Th1 inhibition.

Our study suggests that TLR2 on DCs plays an important role in immune tolerance. Smith M.F. et al., however, showed that TLR2 on epithelial cells activates inflammatory mediators [14]. Thus, global TLR2 deficiency would, on the one hand, decrease immune tolerance resulting in more severe gastritis and, on the other hand, decrease epithelial inflammatory responses resulting in less severe gastritis. Enhanced gastric immunopathology observed in *H. pylori*-infected TLR2KO mice (Figures 4 and 5) indicates that the impact of total TLR2 deficiency is greater on immune cells than on epithelial cells. This is consistent with a report by Sayi A et al. that *Helicobacter* infection in mice deficient in MyD88 (a TLR2 adaptor protein required for downstream signaling) developed accelerated gastric histopathology [33]. They also found that TLR2 signaling mediates *Helicobacter* –stimulated B cell induction of T regulatory-1 cells. Thus, our finding supports further the tolerogenic role of TLR2 in *H. pylori* immune escape.

We also found that the helper T cell priming by BMDCs stimulated with synthetic TLR2 ligand, Pam3Cys, is different compared to T cell priming by *H. pylori*–stimulated BMDCs. Pam3Cys–stimulated BMDCs induced higher Th17/Th1 response and lower Treg response, compared to *H. pylori*–stimulated BMDCs suggesting the *H. pylori*–induced TLR2-mediated Th17/Treg responses are unique to *H. pylori*. We speculate that *H. pylori*-specific TLR2 ligand may signal through TLR2 on BMDCs to promote immunologic tolerance. This concept was described recently by Round JL et al. showing a symbiotic factor (polysaccharide A) of *Bacteroides fragilis* signals through TLR2 on Foxp3+ Tregs to promote immunologic tolerance [34]. Similar findings was reported in another study showing that *Staphylococcus aureus* controls adaptive immune responses through IL-10 and TLR2 [35].

A question remains whether Th17 induction in TLR2-sufficient mice plays a role in *H. pylori* immune escape. Th17 is essential for inducing neutrophil-attracting chemokines leading to *H. pylori* clearance [36]. Hlizler et al. showed that Th1 or Th17 are required for vaccine-induced *H. pylori* clearance [24]. While it is clear that an early Th17 response is important in the control of *H. pylori* infection in the setting of vaccine-induced immunity [37], it plays an antiinflammatory role in chronic *H. pylori* infection by suppressing the Th1 response [38]. Our study provides further evidence that Th17 response in chronic *H. pylori* infection may reduce the protective Th1 response and defective TLR2 induction of Th17 contributes further to the unopposed Th1 response and reduces *H. pylori* colonization. Of note, our findings have particular clinical relevance as most individuals infected with *H. pylori* acquire the infection during childhood and are chronically infected.

In conclusion, our findings show that the absence of TLR2 signaling during *H. pylori*- DC interaction reduces Treg and Th17 priming and enhances Th1 induction by DCs. In vivo, *H. pylori* infection in TLR2-KO mice exhibits reduced bacterial density and increased *H. pylori*–specific Th1 response. Our study indicates that TLR2 signaling is required for *H. pylori* survival, mediating DC Treg and Th17 priming, which is critical for *H. pylori* host immune escape. This presents a novel mechanism in the pathogenesis of *H. pylori* infection. TLR2 may be an important target in the modulation of the host response to *H. pylori*.

## Supporting Information

Figure S1 **Synthetic TLR2 ligand versus *H. pylori*-stimulated BMDC priming of helper T cell responses.** BMDCs were pulsed with PBS, Pam3Cys (100ng/mL), or live H. pylori ((multiplicity of infection, 10:1) for 18 h and cocultured with naive syngeneic splenocytes (1 × 106 cells/well) for 72 h at a splenocyte-to-DC ratio (10:1). T cells were labeled with FITC-conjugated CD4 and intracellular expression of Foxp3, IL-17A, and IFNγ wee measured by flow cytometry. Representatives of three separate experiments are shown.
(TIF)
