# Supplementary figures and images for: TLR2 Mediates Helicobacter pylori–Induced Tolerogenic Immune Response in Mice
Source: PLoS One. 2013 Sep 13;8(9):e74595. doi: 10.1371/journal.pone.0074595 (PMC3772856; doi:10.1371/journal.pone.0074595)

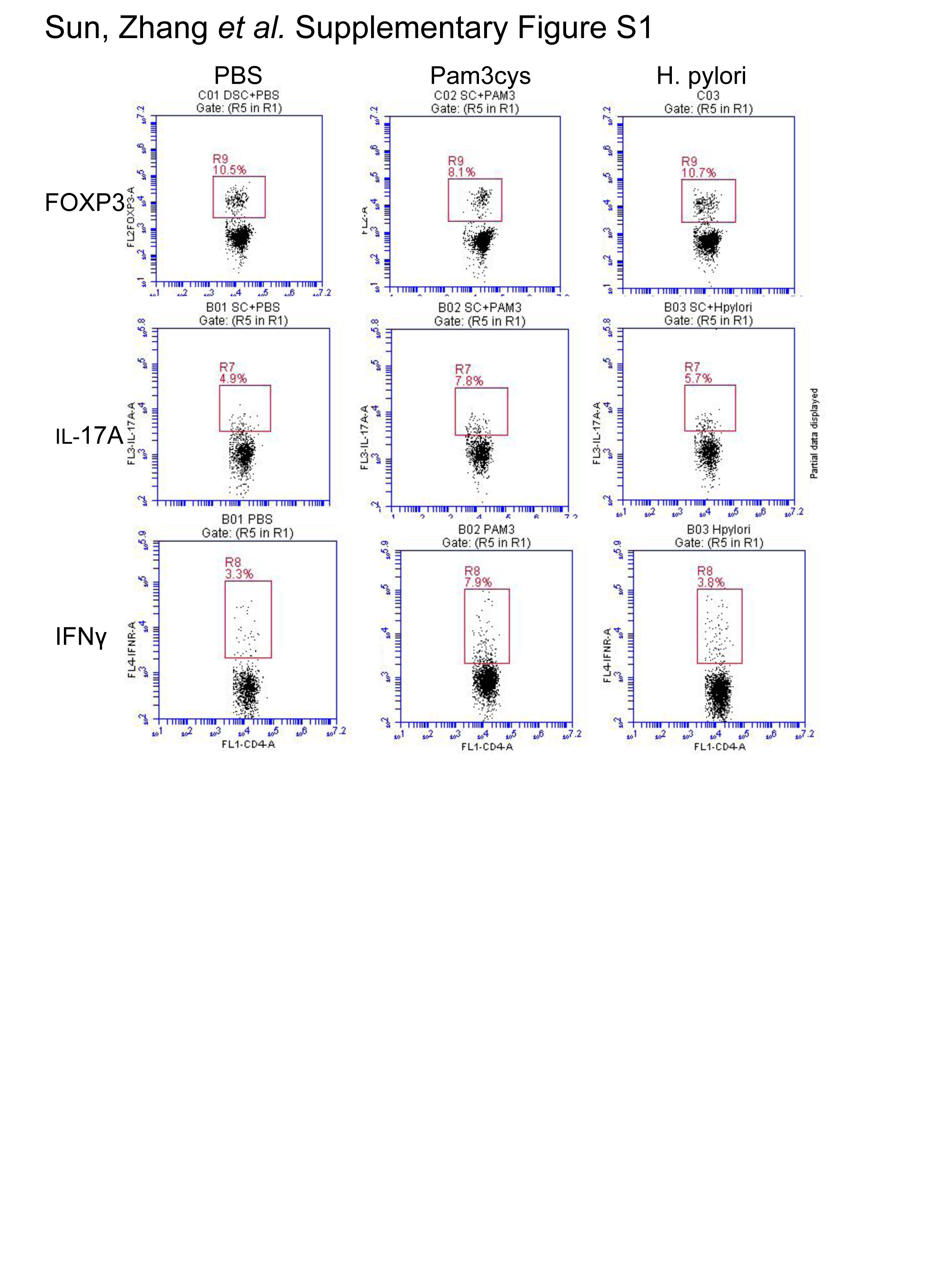

Supplement: Figure S1 — Synthetic TLR2 ligand versus H. pylori-stimulated BMDC priming of helper T cell responses. BMDCs were pulsed with PBS, Pam3Cys (100ng/mL), or live H. pylori ((multiplicity of infection, 10:1) for 18 h and cocultured with naive syngeneic splenocytes (1 × 10^6^ cells/well) for 72 h at a splenocyte-to-DC ratio (10:1). T cells were labeled with FITC-conjugated CD4 and intracellular expression of Foxp3, IL-17A, and IFNγ wee measured by flow cytometry. Representatives of three separate experiments are shown. (TIF) [file pone.0074595.s001.tif]
